# Prevalence, clinical features and prognosis of malignant solid tumors in infants: a 14-year study

**DOI:** 10.17305/bjbms.2020.5121

**Published:** 2021-10

**Authors:** Tian Zhi, Wei-Ling Zhang, Yi Zhang, Yi-Zhuo Wang, Dong-Sheng Huang

**Affiliations:** Department of Pediatrics, Beijing Tongren Hospital, Capital Medical University, Beijing, China

**Keywords:** Infants, malignant solid tumors, clinical features, prognosis

## Abstract

The onset of malignant solid tumors in infants is insidious and difficult to diagnose in time. The purpose of this study was to provide a theoretical basis for clinical diagnosis by performing a retrospective analysis of the data in the past 14 years. In this study, we retrospectively collected the clinical data of infants aged 0–12 months with malignant solid tumors in Beijing Tongren Hospital Affiliated to Capital Medical University from May 2005 to May 2019. The epidemiology, clinical characteristics, treatments, and prognoses were statistically analyzed. A total of 496 infants (294 males and 202 females) with malignant solid tumors were evaluated. The main period of onset was 1–11 months. The most common tumor was retinoblastoma (RB; 51.8%), followed by hepatoblastoma (HB; 26.6%), neuroblastoma (NB; 10.5%), rhabdomyosarcoma (RMS; 3.4%), malignant renal tumors (3.2%), infantile fibrosarcoma (IFS; 1.6%), malignant teratoma (1.2%), Ewing’s sarcoma (ES; 0.8%), medulloblastoma (MB; 0.4%), and inflammatory myofibroblastic tumor (IMT; 0.4%). The median follow-up time was 32 months (range, 2–162 months). At 1, 3, and 5 years, the overall survival rates of all the patients were 97.3%, 89.2%, and 81.1%, respectively, and the event-free survival rates were 94.7%, 84.8%, and 75.8%, respectively. In conclusion, as a special colony, malignant solid tumors in infants are complex, heterogeneous, and relatively rare. The prognosis of RB, HB, NB, RMS, malignant renal tumors, IFS, malignant teratoma, ES, MB, and IMT were excellent owing to timely diagnosis and rational treatment.

## INTRODUCTION

With the improvement of modern medical technology, the prognosis of patients with tumors has greatly improved, but tumors are still the second most frequent cause of childhood death after accidental injury [1]. In recent years, malignant solid tumors in infants have attracted the attention of domestic and international scholars [2]. According to the data of the Surveillance Epidemiology and End Results (SEER) in the United States, the incidence rate of malignant tumors in infants has gradually increased in the past 30 years, and malignant solid tumors account for more than two-thirds of infant malignant tumors [3]. Most infant tumors occur within the first 28 days of life, and the process is fundamentally different from that of most other pediatric and adult tumors [4]. In addition, the age of onset of malignant solid tumors in children has been reported to be getting younger, especially in the first year after birth [5]. Fetuses were found to have space-occupying lesions on B-ultrasonography that were confirmed to be malignant solid tumors by pathological examination after birth [4].

Malignant solid tumors in infants are different from tumors of old children, and knowledge gained from treating older children cannot be used in infants [6]. The histological type of infant tumors is mostly embryonal or germ cell tumor [7]. Owing to the growth and development characteristics of infants, the tumor pathogenesis, treatment response, and prognosis are significantly different from those in older infants [8, 9]. Furthermore, the onset of infant malignant solid tumors is insidious and difficult to diagnose in time. In China, few studies have been conducted on infant malignant solid tumors.

Therefore, we aimed to retrospectively analyze the clinical characteristics, treatment, and outcomes of some infant malignant solid tumor involvements in this study and provide valuable information for diagnosis and treatment.

## MATERIALS AND METHODS

### Patients

In this single-center retrospective study, we enrolled 496 infants with malignant solid tumors who were admitted at the Pediatric Department, Beijing Tongren Hospital Affiliated to Capital Medical University, between May 2005 and May 2019. The study was approved by the ethics committee of the Beijing Tongren Hospital Affiliated to Capital Medical University (ethical batch No. TRECKY 2019-033). All relevant examinations and treatments were performed after obtaining the informed consent of the patients’ guardians.

Infants aged ≤12 months were eligible for this study if they were diagnosed as having a malignant solid tumor by pathological examination or clinical diagnosis. Several types of malignant solid tumor were included as follows: retinoblastoma (RB), hepatoblastoma (HB), neuroblastoma (NB), rhabdomyosarcoma (RMS), malignant renal tumors (nephroblastoma, clear cell sarcoma of kidney [CCSK], and renal RM), infantile fibrosarcoma (IFS), inflammatory myofibroblastic tumor (IMT), germ cell tumors (TGCTs), Ewing’s sarcoma (ES), and medulloblastoma (MB). Patients with unclear clinical or pathological diagnosis, non-infancy, and non-new-onset solid tumors were excluded from the study.

### Diagnosis and treatment regimes

The primary tumor foci, tumor markers, and imaging examinations were considered as clinical diagnostic methods. The International Intraocular Retinoblastoma Classification (IIRC) was used for RB staging [10]. The diagnosis of HB was based on the Children’s Hepatic Tumors International Collaboration (CHIC) [11] and Pre-treatment Extent of Tumor (PRETEXT) risk stratification systems, pathological diagnosis, and alpha-fetoprotein level (AFP). The NB stage was determined using the International Neuroblastoma Staging System (INSS) [12]. The RMS Intergroup Rhabdomyosarcoma Study (IRS) staging system was used as the basis for the RMS staging [13]. Genetic testing was used for assessing genetic susceptibility.

Surgery, chemotherapy, radiotherapy, interventional therapy, and new treatments were adopted in our study. The formulation of a chemotherapy regimen was based on the advice of the Children’s Oncology Group and the experience of our hospital. Presurgical chemotherapy was sustained for 3–5 cycles; and postsurgical chemotherapy, for 4–6 cycles. One cycle of chemotherapy lasted for 21–28 days. The conventional first-line chemotherapy regimens were recommended for most patients. For patients with poor first-line chemotherapy prognosis, individualized chemotherapy regimens could be used. Laser treatment, vitrectomy, intraocular injection, and interventional therapy were recommended for infants with refractory solid tumors.

### Efficacy evaluation and follow-up

Patient follow-up was conducted until May 2020, which was completed by returning to the hospital for reexamination or telephone interview. A survival analysis was used to evaluate overall survival (OS) and event-free survival (EFS). OS was defined as the time from admission to death due to any causes. EFS was defined as the time from admission to the first tumor recurrence/metastasis or death due to any reason.

### Statistical analyses

All statistical analyses were performed using the SPSS 19.0 software (SPSS Institute. IL.USA). Normal distribution data were expressed as mean ± standard deviation (SE); and non-normal distribution data, as median and interquartile range (IQR). Survival data were analyzed using the Kaplan-Meier survival analysis method. Statistical significance was set at *p* < 0.05.

## RESULTS

### Patient characteristics

**FIGURE 1:**
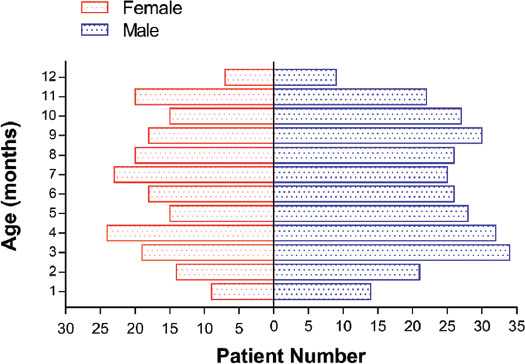
Sex and age distribution of solid tumors in infants.

From May 2005 to May 2019, 496 infants with diagnosed infantile malignant solid tumors in our center were enrolled in this analysis. Of the patients, 294 (59.3%) were male and 202 (40.7%) were female, with a median age of onset of 6.23 months (range, 0–12 months). In most patients, the age of onset was between 1 and 11 months (Figure 1).

The most common type of tumor was RB (257/496, 51.8%), followed by HB (132/496, 26.6%), NB (52/496, 10.5%), RMB (17/496, 3.4%), malignant renal tumors (16/496, 3.2%), IFS (8/496, 1.6%), malignant teratoma (6/496, 1.2%), ES (4/496, 0.8%), MB (2/496, 0.4%), and IMT (2/496, 0.4%). Nine patients had a family history of malignant tumors in their lineal consanguinity. During pregnancy, 5 patients (1.0%) were found to have space-occupying lesions on B-ultrasonography, which were confirmed to be malignant solid tumors by pathological examination after birth.

### Prevalence

**FIGURE 2:**
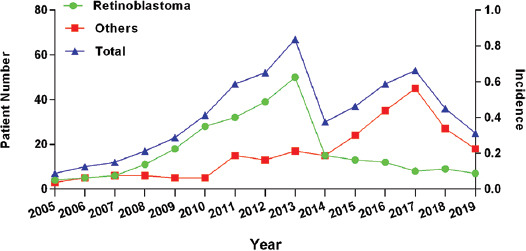
Epidemiology of malignant solid tumors in infants.

As shown in Figure 2**,** over the past 14 years, the number of RB cases increased every year and peaked in 2013 (50/496, 10.1%). The incidence of other types of malignant solid tumor also showed an increasing trend every year, but the peak occurred slightly later than that in the RB cases and was concentrated in 2017 (45, 9.1%). In terms of patient distribution, the enrolled infants were widely distributed, mainly in north China. The top 4 regions were Hebei (65, 13.1%), Shandong (58, 11.7%), Henan (41, 8.3%), and Beijing (38, 7.6%).

### Baseline characteristics, treatments, and outcomes

The baseline characteristics and treatments of all the following diseases are shown in Table 1.

**TABLE 1:**
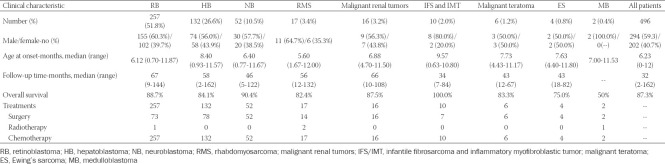
Baseline characteristics and treatments of different tumor types.

### Retinoblastoma

RB was the most common tumor in the enrolled patients in this study (257 cases, 51.8%), including newborns (22, 8.6%) and infants aged 2–12 months (235, 91.4%). Leukocoria and yellow-white pupillary reflection (80.9%) were the most common first symptoms. Five patients had a family history of RB, of whom 2 had intraocular space-occupying lesions during the fetal period. Unilateral (118, 45.9%) and bilateral RBs (139, 54.1%) were also found. In the 257 patients with RB, 396 eyes were affected by intraocular RB (225, 358 eyes, 90.4%), extraocular RB (25, 31 eyes, 7.8%), and metastatic RB (7, 7 eyes, 1.8%). The neonates with metastatic RB were predominant. In accordance with the IIRC [10], intraocular RBs were divided into stages A–E, with stage D (172, 43.4%) being the most prevalent. Among the extraocular RBs, 20 cases invaded the optic nerve and/or the optic nerve stump, and 5 invaded the orbital tissues such as the eye muscles. The patients with metastatic RB had unilateral lesions, and slightly more patients had the RB in the left eye (5 cases), including 1 patient with bone metastasis, 3 patients with intracranial metastasis, and 3 patients with cerebrospinal fluid metastasis.

With regard to treatment, 73 patients underwent surgery, 1 underwent radiotherapy, and 257 underwent chemotherapy (carboplatin [CAR]/etoposide [ETO] or teniposide [TEN]/vincristine [VIN]). In addition, 44 patients received new treatments (laser treatment, 12; vitrectomy, 23; and intraocular injection, 9). Thirty-nine patients underwent transcatheter ophthalmic artery chemotherapy. A total of 114 eyeballs were removed from 396 affected eyes and diagnosed with RB. The eye-preserving rate was 71.2%.

The OS rate of the patients with RB was 88.7%, with 29 deaths (intraocular RB, 11; extraocular RB, 11; and metastatic RB, 7). The OS rates of the newborns and infants aged 2–12 months were 86.4% and 88.9%, respectively. The survival rates of the patients with intraocular, extraocular, and metastatic RB were 95.1% (214/225), 56.0% (14/25), and 0 (7/7), respectively, with statistically significant differences between the patient groups (*p* = 0.015). This suggests that earlier tumor stage correlated with better prognosis.

### Hepatoblastoma

Of the 132 patients with HB, 8 newborns and 124 infants aged 2–12 months were enrolled (132/496, 26.6%). At the first diagnosis, abdominal distension was found to be the most prevalent (75.0%), followed by poor appetite, vomiting, and diarrhea. In this study, 2 patients had a relevant family medical history. Three patients had liver space-occupying lesions during pregnancy (gestational weeks: 32–36 weeks) and were pathologically diagnosed as having HB after birth; all of them had low birth weights.

All the patients were diagnosed pathologically and on the basis of their AFP levels (reference interval, 0–20 ng/ml). In our study, the mean AFP level of the 132 patients with HB was 127.41 ± 7.23 μg/ml (range, 0.04–484.0 μg/ml) at the first diagnosis. In accordance with the CHIC risk stratification system[11], all pathological tissues were classified as epithelial (76/132, 57.6%) or mixed (56/132, 42.4%). The epithelial type included fetal (51/76, 67.1%), embryonic (21/76, 27.6%), giant beam (2/76, 2.6%), and small cell undifferentiated types (2/76, 2.6%). All the infants were divided into stage I (8/132, 6.1%), stage II (45/132, 34.1%), stage III (72/132, 54.5%), and stage IV (7/132, 5.3%), referring to the PRETEXT risk stratification system[14]. Forty-five patients had a distant metastasis (newborns/infants aged 2–12 months, 5/40), and the most common was lung metastasis (39/45, 86.7%), followed by intracranial metastasis (6 cases), bone metastasis (4), right atrial tumor thrombus (2), intestinal and mesenteric metastasis (1), and intraspinal metastasis (1). This study included tumor rupture (5/132, 3.8%) and multiple intrahepatic lesions (26/132, 19.7%).

All the patients received platinum-based chemotherapy, including cisplatin (CIS)/fluorouracil (FLU)/VIN, CIS/FLU/VIN/doxorubicin (DOX), CIS/DOX, CAR/DOX, and ifosfamide (IFO)/CAR/ETO. Seventy-eight patients received surgery, with a complete resection rate of 59.1%. After the first operation, 16 patients underwent a second operation due to recurrence, and 26 underwent resection of the metastatic tumor. One patient had a complete remission after liver transplantation. Eight patients were administered with bevacizumab, with a therapeutic efficacy of 88%. Patients with PRETEXT stage III or IV who still had an unresectable lesion after standard treatment were assigned to receive an interventional therapy. Among these patients, 21 died, with an OS rate of 84.1%. The OS rates of the newborns and infants aged 2–12 months were 50% and 86.3%, respectively.

### Neuroblastoma

Fifty-two patients had NB (52/496, 10.5%), including newborns (6, 11.5%) and infants aged 2–12 months (46, 88.5%). Forty cases of primary tumors occurred in the adrenal gland, most of which had a unilateral onset; only one case was a bilateral adrenal space-occupying lesion. The primary tumors in 12 cases originated from the mediastinum. In accordance with the INSS, all the infants were divided into stage I (3/52, 5.8%), stage II (9/52, 17.3%), stage III (15/52, 28.9%), stage IV (14/52, 26.9%), and stage IVs (11/52, 21.1%) in our study [12]. Furthermore, 25 patients had distant metastasis, including lymphatic (9, 34.6%), hepatic (6, 21.1%), osseous (4, 17.3%), intraspinal (3, 13.7%), and subcutaneous metastases (3, 13.3%).

All the patients received surgery and chemotherapy. The chemotherapy regimens were cyclophosphamide (CYC)/DOX/VIN, ETO/CIS, CYC/VIN/CIS/DOX or ETO, and CYC/topotecan (TOP). Nine patients underwent a second surgery due to recurrence or metastasis. Two patients with increased GD-2 expression levels received a cellular immunotherapy with CAR-T.

The mean neuron-specific enolase level of the 52 patients with NB was 103.9 ± 52.7 ng/ml (range, 22.4–370 ng/ml; reference interval, 0–16.3 ng/ml). Moreover, 7 patients (7/36, 19.4%) carried the positive variant in the *N-myc* gene with a survival rate of 71.4% (5/7). Five patients died, with an OS rate of 90.4% (newborns, 83.3%; infants, 91.3%).

### Rhabdomyosarcoma

A total of 17 patients (17/496, 3.4%) with RMS were included. One patient had a relevant family medical history. Most RMS cases (14/17, 82.4%) originated in the head and neck, preponderant in the orbital space. The most common pathological type was embryonic tumor (15/17, 88.2%). In accordance with the IRS staging system, all the patients were divided into stage I (2/17, 11.8%), stage II (4/17, 23.5%), stage III (6/17, 35.3%), and stage IV (5/17, 29.4%).

Seventeen patients underwent surgery, 14 underwent radiotherapy, and 2 underwent chemotherapy (DOX/VIN/CYC/CIS, IFO/VIN/ETO, and dactinomycin [DAC]/ETO/VIN). Owing to the younger age of the group, they were not treated with radiotherapy, only surgery and chemotherapy, after the initial diagnosis. Two patients were treated with radiotherapy (seed implantation and external irradiation) after recurrence but eventually died. Three deaths were reported, with an OS rate of 82.4%.

### Malignant renal tumors

In our analysis of 16 infants (16/496, 3.2%) with malignant renal tumors, one patient had a relevant family medical history. The pathological types were as follows: nephroblastoma (14/16, 87.5%), renal RMS (1/16, 6.2%) and CCSK (1/16, 6.2%). No distant metastasis was reported in this population. Patients with distant metastasis were treated with surgery and chemotherapy. The chemotherapy regimens were VIN, VIN/DAC or pirarubicin (PIR), CYC/VIN/PIR/ETO, and CAR/IFO/ETO. After the first operation, 3 patients underwent nephrectomy due to tumor recurrence. The OS rate of the malignant renal tumors was 87.5%.

### IFS and IMT

Ten patients were enrolled. Eight patients (8/10, 80.0%) were diagnosed as having IFS, including hand/foot, back, and retroauricular masses. In addition, 3 patients tested positive for the *EVT6* gene by fluorescence in situ hybridization testing. Two patients (2/10, 20.0%) had IMTs. The primary tumors were located in the abdominal mesentery. All the patients underwent chemotherapy, and 70% of them underwent a surgery. The chemotherapy regimens included vindesine (VIND)/CYC/DAC and VIND/PIR/CYC/CIS. No death was reported, and the OS was 100%.

### Malignant teratoma

Six patients (6/496, 1.2%) with malignant teratoma were enrolled. The primary tumor had gonadal (left testis) and extragonadal origins, mostly in the sacrococcygeal. One of the patients had an S3 level intraspinal metastasis and died due to tumor progression. All the patients received surgery and chemotherapy, which included VIN/bleomycin (BLE)/CIS and BLE/ETO/CIS. The OS rate of the patients with malignant teratoma was 83.3%.

### Ewing’s sarcoma

Among the 4 patients with ES, 2 underwent a *EWSR1* gene test, but both tested negative. The primary tumor in the 4 patients originated from the eye socket, nasal wing, and pelvic cavity. All the patients underwent surgery and chemotherapy, with an OS rate of 75%.

### Medulloblastoma

Two patients (2/496, 0.4%) were enrolled, and both had a classic MB. The primary site was the fourth ventricle. No distant metastasis was found. Both patients received effective treatments such as surgery and chemotherapy, and one received radiotherapy. In addition, intrathecal injection is also an effective adjuvant therapy. One patient died of respiratory and cardiac arrest due to tumor compression of the brain stem. The OS rate was 50%.

### Survival analysis

**FIGURE 3:**
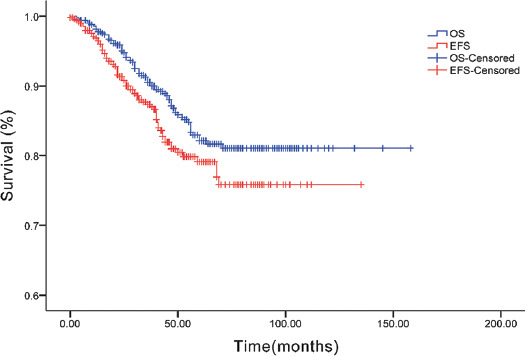
Overall survival and event free survival of 496 infants with malignant solid tumors.

**FIGURE 4:**
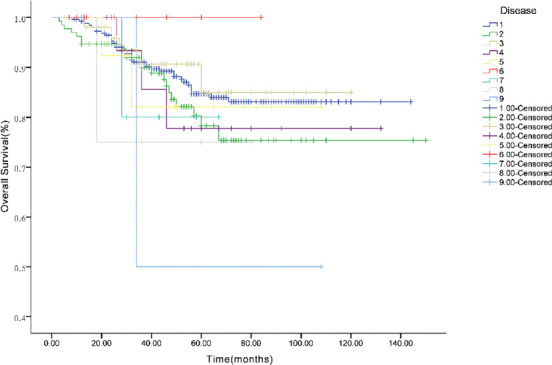
Overall survival curve of different tumor types. 1 retinoblastoma (RB), 2 hepatoblastoma (HB), 3 neuroblastoma (NB), 4 rhabdomyosarcoma (RMS), 5 malignant renal tumors, 6 infantile fibrosarcoma and inflammatory myofibroblastic tumor (IFS/IMT), 7 malignant teratoma, 8 Ewing’s sarcoma/primitive neuroectodermal tumor (ES/PNET), 9 medulloblastoma (MB).

**FIGURE 5:**
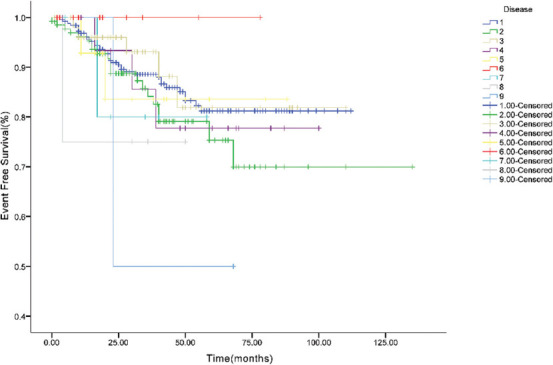
Event free survival curve of different tumor types. 1 retinoblastoma (RB), 2 hepatoblastoma (HB), 3 neuroblastoma (NB), 4 rhabdomyosarcoma (RMS), 5 malignant renal tumors, 6 infantile fibrosarcoma and inflammatory myofibroblastic tumor (IFS/IMT), 7 malignant teratoma, 8 Ewing’s sarcoma/primitive neuroectodermal tumor (ES/PNET), 9 medulloblastoma (MB).

As of May 2019, the median follow-up time was 32 months (range, 2–162 months). Among the 496 patients, 63 died, with a survival rate of 87.3%. The Kaplan-Meier survival analysis showed that the 1-, 3-, and 5-year OS rates of the included patients were 97.3%, 89.2%, and 81.1%, respectively. The 1-, 3-, and 5-year EFS rates were 94.7%, 84.8%, and 75.8%, respectively (Figure 3). As shown in Figures 4 and 5, we analyzed the OS and EFS for each disease. The estimated 3-year EFS rates of the patients with RB, HB, NB, RMS, malignant renal tumors, malignant teratoma, ES/primitive neuroectodermal tumor (PNET), and MB were 88.0% ± 2.2%, 88.0% ± 2.2%, 86.9% ± 3.5%, 85.2% ± 6.0%, 77.8% ± 11.4%, 81.6% ± 10.8%, 80.0% ± 17.9%, 75.0% ± 21.7%, and 50.0% ± 35.4%, respectively.

## DISCUSSION

To our knowledge, this is the first long-term study of infant malignant solid tumors in China. We report our observations over the past 14 years and found that malignant solid tumors in infants are complex and changeable. However, with timely diagnosis and rational treatment, the patient prognosis was excellent.

Infant tumors are mostly embryonic-derived tumors, and their pathogenesis involves developmental biological, genetic, environmental, and other factors [15]. Genetic predisposition has been shown to be one of the important causes of infant tumors [16]. RB, NB, and HB all have obvious genetic susceptibility. N-myc, WT1, and RB1 genes are associated with NB, nephroblastoma, RB, and other tumors, respectively. In the process of fetal development, activation or inhibition of these genes can lead to the impairment of embryo development and even carcinogenesis [17-19]. In our study, 9 infants (1.8%) had a family history of malignancies in their immediate family, including 5 cases of RB, 2 cases of HB, 1 case of RMS, and 1 case of nephroblastoma.

A report analyzed the incidence of childhood cancers in the past 29 years, The incidence in males and females were 0.155‰ and 0.136‰, respectively [20]. Similarly, in this study, most cases occurred in males. The infant tumors were mainly embryonic solid tumors. Another institution demonstrated that the top three diseases with the highest incidence rates were RB (44%), leukemia (19%), and NB (10%) [21]. Our result showed that RB accounted for 51.8% of all malignant solid tumors in infants, higher than the previous report. The possible reason was that our hospital is specialized in ophthalmology. Many patients with infant malignant solid tumors attend our hospital for treatment before 2013. However, after 2013, with the changes in the treatment patterns in our hospital, maturity of treatment technology in local hospitals, and support of the medical insurance policy, most patients prefer to choose local hospitals for treatment. The incidence of other solid tumors also showed an increasing trend. In addition, we found that the number of HB cases was lower than that of RB cases (26.6%), but the incidence rate was significantly higher than those reported in other articles. This is consistent with the study of Hung et al. in which the incidence rate of HB in the Taiwan region of China was 2–5 times higher than those in European and American countries [15]. We speculate that gene variation may be a risk factor of HB in the Han nationality [22].

In terms of regional distribution, the patients’ residences cover >30 provinces and cities in China. However, owing to the geographical location of our center, most patients were still mainly from North China. In this study, the RB cases in infancy were mostly intraocular (90.4%). Among these cases, stage D RB (80.3%) was dominant. A few patients with metastatic RB died. A previous study showed that stage E (80.3%) was preponderant in the intraocular RBs of older children [23]. We found that the RB stage in the infants was lower than that in the older children, and metastatic RB was rare. We speculated that the reason for this difference may be related to the young age of onset, the early detection of the disease, and the tumor having not yet metastasized. However, once the tumor breaks through the eyeball and has a distant metastasis, the prognosis is often poor.

Künkele et al reported that the current globe salvage rate of RB could reach 62%–75% (IIRC stages A–C, 100% and stages D and E, 50%–80%) worldwide [24]. The eye salvage rate in our study was 71.2%, including 100% for stages A, B, and C, 80.2% for stage D, and 44.8% for stage E, consistent with other literature reports.

Through a statistical analysis of great data, Piotr Czauderna reported that HB in infancy was mainly of pure embryonic type with relatively good differentiation and better EFS[25]. From the distribution of the pathological tissues in this study, the major type of HB in infancy was the epithelial type. Among the epithelial types, the fetal type had the best prognosis, consistent with the literature report. According to the data from the Society of Pediatric Oncology Liver Tumor Study Group, the PRETEXT staging can predict the resectability of tumors, which is crucial for prognosis [26]. Data from this study showed more infants with PRETEXT stage III and fewer infants with stage IV. The complete tumor resection rate was 59.1%, close to the international level [27]. Systemic metastasis can occur in the early stage of HB, as in older children with HB. The lung is the most common site of metastasis [28]. Our result shows that among the 45 patients with distant metastasis, 86.7% had lung metastasis, consistent with the previous report.

NB originates from the adrenal medulla to the sympathetic nervous system. The prognosis of infants with NB is better than that of older children with NB, and even some children with stage IVs NB can have spontaneous regression in the first few months after birth [29]. The positive variant in the *N-myc* gene is closely related to the poor prognosis of NB. The incidence of the positive variant in *N-myc* in infants with NB is approximately 10%, with a survival rate of 34.3% [17, 30]. In this study, the incidence accounted for 19.4% of all cases (7/36), and the survival rate was 71.4%, which was higher than that reported in the literature. It may be related to the small sample size.

Soft tissue sarcoma in children is composed of a series of malignant connective tissues, and RMS accounts for more than half of them. RMS could be found in the head, neck, limbs, genitourinary system, and other parts [26]. In this study, 82.4% of the primary tumors in the 14 cases originated in the head and neck, which was higher than that reported in the literature [31]. The possible reason was that the level of the otorhinolaryngology, head and neck surgery department in our hospital ranked among the top in China, so the source of patients was relatively large. The histological classification of infant RMS was mainly embryonal, and the prognosis was better than those of other types [32]. In these data, 88.2% of the RMS cases were embryonal, consistent with relevant reports. IFS occurs at an early age, with 35% of cases occurring after birth, but the long-term survival rate is close to only 90% [33]. Eight children who were included in this study had earlier onset ages than those reported for other diseases. We treated them according to the treatment protocol of the European Pediatric Soft Tissue Sarcoma Study. All the patients survived and achieved excellent prognoses. In this study, nephroblastoma accounted for 87.5%, with no distant metastasis, and its prognosis was better than those of CCSK and renal RMS, which supported the previous publications [34].

On the basis of the international classification of pediatric cancer, infant malignant tumors are extremely aggressive high-grade tumors, but their prognosis is much better than that of malignant tumors in older children and adults [35]. Alfar et al. evaluated 615 newborns with malignant tumors from the SEER database in the United States and found that the 5-year OS rate of patients with malignant solid tumors was 71.2% [36]. One French study showed that the 5-year OS rate of newborns with malignant solid tumors was 83.8% [4]. In this study, the 5-year OS rate of the infants with malignant solid tumor was 81.1%, which was basically consistent with the reference reports.

In this study, we found the OS rates of RB, NB, malignant renal tumors, and IFS were >85%. Nevertheless, in some low-income countries, the OS rates of infants with RB ranged from 30% to 60% [37]. The reason may be that the patients were given good eye service in our hospital. A long-term survival result based on 409 patients showed that the OS rate of pediatric patients with RMS was 33%, which was lower than that in our research [38]. We speculate that the considerable disparity resulted from the small sample size. With the same reason, we cannot adequately compare newborns with infants aged 2–12 months. We only found that the proportion of neonatal tumors was higher than that in a previous study and that the OS rates of neonates with RB and HB were lower than those of older infants [39]. Newborns have an immature physiology, and their hematopoietic and immune systems are not yet fully developed. Moreover, their response to therapy is unpredictable [40]. Thus, a larger sample size is needed to fully elucidate the difference in clinical features and outcomes among newborns and older infants.

This study also has several limitations. First, the sample size of the patients with malignant solid tumors was small. The sample size was also not enough to compare newborns with older infants. Second, the follow-up period in some patients was relatively short. Thus, in future research, large-scale investigations with long-term follow-up are needed.

## CONCLUSION

As a special colony, malignant solid tumors in infants are complex, heterogeneous, and relatively rare. The prognosis of malignant solid tumors in infants is often better with comprehensive treatment. However, accurate diagnosis, evaluation, and treatment of these tumors are still challenging. This study provides evidence for the diagnosis and treatment of malignant solid tumors in infants. However, an infant tumor database must be established through a joint collaboration of multiple centers to make concerted efforts to better improve the survival and prognosis of infants with malignant solid tumors.
